# Curcumin Co-Encapsulation Potentiates Anti-Arthritic Efficacy of Meloxicam Biodegradable Nanoparticles in Adjuvant-Induced Arthritis Animal Model

**DOI:** 10.3390/biomedicines11102662

**Published:** 2023-09-28

**Authors:** Bilal Aslam, Asif Hussain, Muhammad Naeem Faisal, Zia-ud-Din Sindhu, Rifat Ullah Khan, Ibrahim A. Alhidary, Shabana Naz, Vincenzo Tufarelli

**Affiliations:** 1 Institute of Physiology and Pharmacology, University of Agriculture Faisalabad, Faisalabad 38040, Pakistan; asifhussain072@gmail.com (A.H.); m.naeem.faisal@uaf.edu.pk (M.N.F.); 2 Department of Pharmacy, Riphah International University, Faisalabad 38000, Pakistan; 3 Department of Parasitology, University of Agriculture Faisalabad, Faisalabad 38040, Pakistan; sandhu@uaf.edu.pk; 4 College of Veterinary Sciences, Faculty of Animal Husbandry and Veterinary Sciences, The University of Agriculture, Peshawar 25130, Pakistan; 5 Department of Animal Production, College of Food and Agriculture Sciences, King Saud University, Riyadh 11421, Saudi Arabia; iahidary@ksu.edu.sa; 6 Department of Zoology, Government College University, Faisalabad 54000, Pakistan; drshabananaz@gcuf.edu.pk; 7 Department of Precision and Regenerative Medicine and Jonian Area (DiMePRe-J), Section of Veterinary Science and Animal Production, University of Bari ‘Aldo Moro’, s.p. Casamassima km 3, 70010 Valenzano, Italy; vincenzo.tufarelli@uniba.it

**Keywords:** curcumin, meloxicam, nanoparticles, rheumatoid arthritis, cytokines, inflammation

## Abstract

This study aimed to evaluate the anti-arthritic activity of curcumin and meloxicam co-loaded PLGA nanoparticles in adjuvant-induced arthritic rats. PLGA nanoparticles encapsulating curcumin (nCur) and meloxicam (nMlx) alone and in combination (nCur/Mlx) were used to characterize zeta size and potential, polydispersity index, encapsulation efficiency (%), compound–polymer interactions (FT-IR analysis), and surface morphology (SEM imaging). In vivo, Complete Freund’s adjuvant-induced arthritic rats were intraperitoneally (i.p.) administered with curcumin, meloxicam, curcumin plus meloxicam, nCur, nMlx, and nCur/Mlx for 28 consecutive days. Results showed that nCur, nMlx, and nCur/Mlx significantly (*p* ≤ 0.05) reduced paw swelling and arthritic score, restored body weight and the immune organ index (thymus and spleen), as well as attenuated serum inflammatory markers (RF, CRP, and PGE2) and oxidative stress parameters (MDA, SOD, and CAT) in adjuvant-induced arthritic rats compared to free compounds. In addition, mono- and dual-compound-loaded nanoparticles significantly (*p* ≤ 0.05) down-regulated pro-inflammatory cytokines (TNF-α, IL-1β, and IL-6), up-regulated anti-inflammatory cytokines (IL-4, IL-10, and IFN-γ), and modulated OPG and RANKL expressions in paw tissue. The aforementioned results were further confirmed through radiological and histopathological examinations. Furthermore, the anti-arthritic effect of nCur/Mlx was notably (*p* ≤ 0.05) enhanced compared to nCur or nMlx alone. In conclusion, the co-nanoencapsulation of curcumin could potentiate the anti-arthritic activity of meloxicam and could provide a novel therapeutic approach for the formulation of nanocarrier pharmaceutical products for the management of arthritis.

## 1. Introduction

Rheumatoid arthritis (RA) is a chronic autoimmune inflammatory disease with serious systemic complications. It primarily affects joints, and the resultant irreversible destructive bone erosion causes musculoskeletal impairment, painful joints, and physical disability. The global prevalence of RA is estimated to be around 0.5 to 1% [1]. Patients with RA have a 50% greater risk of cardiovascular complications, such as atherosclerosis and myocardial infarction, and a 60% higher chance of premature mortality [2,3]. The etiology of RA is still not fully understood. However, various environmental and genetic factors, autoantibodies, oxidative stress, and chronic inflammation are believed to be involved in the pathophysiology of RA [4].

Clinical complications of RA include synovial lining and tendon sheath degeneration in the diarthrodial joints of the hands and feet, which results in joint and bone abnormalities [5]. In RA pathogenesis, the infiltration of macrophages, B-cells, and T-cells, along with resident fibroblasts, aggravates the inflammatory response by overproducing inflammatory cytokines (IL-1β, IL-6, IL-17a, and TNF-α) and prostaglandin E2 (PGE2) and suppressing anti-inflammatory mediators (IL-4, IL-10, and IFN-γ), which ultimately causes synovial hyperplasia, synovitis, and the erosion of cartilage and bone via disturbing the OPG/RANKL balance [6,7]. The production of ROS, as a result of cellular metabolic activities, is also an important factor in the pathogenesis of RA because it may be associated with joint tissue damage. Antioxidants can scavenge ROS successfully and resist tissue damage. However, their imbalance has been postulated in arthritis due to elevated cellular activity and ineffective antioxidant defense systems [8,9].

Despite the fact that there is no well-known cure for RA, medications used to treat RA include corticosteroids, non-steroidal anti-inflammatory drugs (NSAIDs), and disease-modifying anti-rheumatic drugs (DMARDs). These therapies are mostly used to reduce joint pain and inflammation and also slow the progression of disease by interfering with immune system signaling to suppress the inflammatory processes that cause joint degeneration. However, these agents are relatively costly and have a number of unfavorable side effects [10,11]. With the increasing need for medications to treat RA, there has been a rise in research interest in understanding the pathophysiology of RA and developing novel treatments.

Meloxicam is an NSAID that is used to manage pain and inflammation in RA and other inflammatory conditions. It inhibits arachidonic acid metabolism by preferentially inhibiting the cyclooxygenase-2 (COX-2) enzyme and ultimately prevents the synthesis of prostaglandins (PGs) [12,13]. It is also reported that the long-term intake of meloxicam can lead to hepatorenal toxicities [14]. Studies have shown that the side effects of meloxicam may be reduced by using lower dosages or combining natural anti-inflammatory substances [15]. Curcumin, a bioactive compound of *Curcuma longa* L. (turmeric), is known due to its potent anti-inflammatory, antioxidant, and anticancer activities [16,17]. Also, it can be potentially employed to treat oxidative stress-mediated inflammatory diseases [18]. However, the hydrophobic nature and low bioavailability of curcumin and meloxicam are the main limiting factors of their use [19,20].

Nanoparticles (NPs) are particles that range in size from one to several hundred nanometers. The active agents are integrated into the core or adsorbed onto the surface of the NPs. They can be utilized to deliver therapeutic agents, diagnostic-imaging compounds, peptides, proteins, and genes [21]. NPs can be categorized as polymeric NPs, lipid-based NPs, semiconductor NPs, metal NPs, and carbon-based NPs depending on their chemical and physical properties [22]. Polymeric and lipid NPs are the most promising choices due to their composition. In comparison to other types of NPs, the materials utilized to synthesize polymeric and lipid NPs confer biocompatibility, biodegradability, non-toxicity, non-immunogenicity, and a high drug-loading capacity. Polymeric and lipid NPs are the most clinically approved NPs in use [23]. Polymeric NPs, which are used in this study, can improve the therapeutic efficacy of hydrophobic compounds. These NPs can potentially enhance bioavailability and efficacy and reduce the side effects of therapeutic agents [24]. Polyesters such as poly(lactic-co-glycolic) acid (PLGA)-based nanoformulations are highly regarded for their nontoxic, biodegradable, and biocompatible nature. Also, PLGA is preferred in loading a wide range of synthetic/natural compounds alone or combined due to its high loading capacity [25,26].

The Complete Freund’s adjuvant-induced arthritic animal model is frequently employed to evaluate the effectiveness of synthetic or natural agents against RA, as it shares pathophysiological features similar to human RA [27]. Therefore, this study was proposed to evaluate the anti-arthritic effect of curcumin and meloxicam co-encapsulated PLGA nanoparticles (nCur/Mlx) in an adjuvant-induced arthritis rat model.

## 2. Materials and Methods

### 2.1. Reagents

Poly(D,L-lactic-co-glycolic) acid (PLGA, MW 7000–17,000, cas#26780-50-7) from Sigma-Aldrich^®^, St. Louis, MO, USA; polyvinyl alcohol (PVA 1500, MW 44.053 g/mol) from Duksan^®^ Pure Chemicals, Seoul, Korea; curcumin (Cur) from Spectrum^®^, Shanghai, China; meloxicam (Mlx) from Sigma-Aldrich^®^, USA; and Complete Freund’s adjuvant from Invivogen^®^, San Diego, CA, USA, France were purchased. All other reagents and solvents of highest purity were used. These chemicals were purchased under the Higher Education Commission (H.E.C.), Islamabad, Pakistan, grant number 7510.

### 2.2. Synthesis of Nanoparticles

PLGA nanoparticles encapsulating curcumin and meloxicam alone and combined were prepared according to solvent evaporation (single oil-in-water emulsion) method with some modifications [28,29]. PLGA polymer was dissolved in acetone (200 mg/mL) and 2% *w/v* aqueous solution of PVA was prepared. Curcumin and meloxicam alone and in combination were separately dissolved in acetone-dichloromethane solvent mixture (1:2 *v/v*). Organic and aqueous solutions were emulsified with the help of a micro-tip probe sonicator (Sonics & Materials, Newtown, CT, USA) for 30 s at 50 W output power in an ice bath. The excessive organic solvent was removed by continuously stirring and heating each nanoformulation at 37 °C over a magnetic stirrer. Then, formulations were centrifuged at 4 °C for 10 min at 25,000 rpm using a temperature-controlled centrifuge machine, and nanoparticle pellets were separated, washed with Milli-Q deionized water to remove free compounds, and lyophilized. Additionally, supernatants were collected and stored at −20 °C until further analysis.

### 2.3. In Vitro Characterization

#### 2.3.1. Zeta Size, Zeta Potential, and Polydispersity Index

Lyophilized mono and dual-compound-loaded nanoparticles re-suspended in deionized water (ten-folds) were sonicated for 30 s. The zeta size (nm), zeta potential (mV), and polydispersity index (PDI) of each sample were measured using dynamic light scattering (DLS, Zeta Sizer 3000, Malvern^®^, Worcestershire, UK).

#### 2.3.2. Encapsulation Efficiency

The amount of curcumin and meloxicam loaded into polymeric matrix of PLGA mono and dual-compound-loaded nanoparticles was determined indirectly by assessing the collected supernatants [30]. Different concentrations of curcumin and meloxicam were used to prepare the calibration curves, and absorbance was measured using a spectrophotometer of Shimadzu^®^, Kyoto, Japan (λ_max_ for curcumin: 427 nm; λ_max_ for meloxicam: 363 nm). Encapsulation efficiency was calculated using the equation given below:

(1) $$\mathrm{EE}\;(\%)=[\frac{\mathrm{Total}\;\mathrm{compound}\;\mathrm{used}\;(\mathrm{mg})\;–\;\mathrm{Free}\;\mathrm{compound}\;\mathrm{in}\;\mathrm{supernatant}\;(\mathrm{mg})}{\mathrm{Total}\;\mathrm{compound}\;\mathrm{used}\;(\mathrm{mg})}]\times 100$$

#### 2.3.3. FT-IR Analysis

The FT-IR spectrophotometric analysis was carried out to detect the interactions between PLGA polymer and loaded compounds [31]. Each sample in dried solid form was thoroughly mixed with 100 mg of potassium bromide, and high pressure (1000 psi) was applied to prepare spherical disks. The FT-IR instrument of Spectrum Two^®^ (Perkin Elmer, Waltham, MA, USA) was used for analysis, and FT-IR absorption spectra in the range of 4000 to 600 cm^−1^ and at 4 cm^−1^ resolution were acquired using Spectrum^®^ 10.5.3 software.

#### 2.3.4. SEM Imaging

To observe the surface morphology of prepared nanoparticles, one drop of a concentrated aqueous suspension of each sample was placed on a metal grid and allowed to dry at room temperature for 24 h, then coated with gold–palladium and visualized by SEM (JSM5910, Jeol, Akishima, Tokyo, Japan) under an argon atmosphere [32].

### 2.4. In Vivo Study

#### 2.4.1. Experimental Animals

Forty-eight adult healthy female rats of Wistar strain (5 to 7 weeks old; body weight ranging from 160 to 200 g) were acclimatized for one week under standard experimental environment, i.e., 25 ± 1 °C room temperature, 50–60% humidity, and 12 h day/12 h night cycles. Free access to clean drinking water and a standard pellet diet twice a day were provided.

#### 2.4.2. Experimental Design

Rats were randomly allotted to eight groups (six rats per group, *n* = 6). To induce arthritis, a single injection of 100 μL of Complete Freund’s adjuvant was given into the sub-plantar area of the right hind paw of rats [33]. On day 9 (the first day of therapy), curcumin (15 mg/kg b.w.) [34], meloxicam (4 mg/kg b.w.) [35], curcumin (15 mg/kg b.w.) plus meloxicam (4 mg/kg b.w.), and PLGA nanoparticles loaded with equivalent contents of curcumin, meloxicam, and curcumin plus meloxicam were dissolved in normal saline and administered intraperitoneally until the 28th day of therapy, as given below:

GI—Normal group: normal rats received 3 mL/kg b.w. of saline solution (i.p.).
GII—Model group: untreated arthritic rats were administered 3 mL/kg b.w. of saline solution (i.p.).
GIII—Cur group: arthritic rats treated with 15 mg/kg b.w. of curcumin (i.p.).
GIV—Mlx group: arthritic rats treated with 4 mg/kg b.w. of meloxicam (i.p.).
GV—Cur/Mlx group: arthritic rats treated with 15 mg/kg b.w. of curcumin plus 4 mg/kg b.w. of meloxicam (i.p.).
GVI—nCur group: arthritic rats treated with PLGA nanoparticles encapsulating 15 mg/kg b.w. of curcumin (i.p.).
GVII—nMlx group: arthritic rats treated with PLGA nanoparticles encapsulating 4 mg/kg b.w. of meloxicam (i.p.).
GVIII—nCur/Mlx group: arthritic rats treated with PLGA nanoparticles co-encapsulating 15 mg/kg b.w. of curcumin plus 4 mg/kg b.w. of meloxicam (i.p.).

#### 2.4.3. Assessment of Polyarthritis

The paw diameter of each rat was measured using a digital micrometer gauge, and the effect of adjuvant injection on paw inflammation was assessed on days 0, 7, 14, 21, and 28, respectively. To assess the degree of arthritis, an arthritic score of 0 to 4 was assigned to both the ipsilateral and contralateral paws. Grade 0 defined the absence of inflammation; grade 1 denoted inflammation of one of the fingers or mild erythema; grade 2 showed inflammation of more than one finger; grade 3 displayed inflammation of ankle; and grade 4 indicated severe arthritic inflammation of the fingers or ankle. The maximal arthritis score of 8 (4 × 2: inflammation of both hind limbs) was set for adjuvant-induced arthritis [36]. In addition, pre- and post-arthritic changes in body weight of each animal were also determined.

#### 2.4.4. Blood and Organ Sampling

After 28 days of therapy, animals fasted overnight, and blood samples were taken through cardiac puncture under the influence of anesthesia (10 mg/kg b.w., i.p. of ketamine and 2 mg/kg b.w., i.p. of xylazine). Samples were transferred into EDTA and gel clot activator tubes. Following blood collection, all animals were decapitated by cervical dislocation and immune organs, including thymus and spleen, were collected. The wet organ weight was immediately determined to compute the immune organ index (%). Tissues from the hind limbs were taken and appropriately preserved for gene expression profiling and histological examination.

#### 2.4.5. Hematological Analysis

A hematology auto-analyzer of Boule Medical AB^®^, (Stockholm Sweden) was used to examine red blood cells (RBCs), hemoglobin (Hb), and white blood cells (WBCs) in whole blood samples preserved in EDTA tubes.

#### 2.4.6. Biochemical Analysis

Blood samples stored in gel clot activator tubes were incubated at 4 °C for 20 min and then centrifuged at 3500 rpm for 15 min at 4 °C using a temperature-controlled centrifuge machine. The ELISA kits were used to determine the serum levels of rheumatoid factor (RF, cat#E1556Ra, BioTech Lab^®^, Beijing, China), C-reactive protein (CRP, cat#0053Ra, BioTech Lab^®^, China), and prostaglandin E2 (PGE2, cat#E0504Ra, BioTech Lab^®^, China) using an auto-analyzer (Multiskan Go^TM^, Thermo-Scientific, Cambridge, UK).

The oxidative stress parameters were determined by assessing malondialdehyde (MDA) levels and activities of superoxide dismutase (SOD) and catalase (CAT) in sera of rats. Serum MDA levels were measured according to the method described by Lorente et al. [37], and values were presented as nmol/mL. The SOD and CAT activities were determined using the previously devised methods of Pervin et al. [38] and Huang et al. [39], and results were expressed in U/mL.

#### 2.4.7. Gene Expression Analysis

The mRNA expressions of TNF-α, IL-1β, IL-6, IL-4, IL-10, IFN-γ, OPG, and RANKL in paw tissue were determined by qRT-PCR. Paw tissues preserved in RNALater^®^ were homogenized, and total RNA was extracted using the TRIzol (Thermo-Scientific^®^, UK) technique. A Nanodrop spectrophotometer was used to quantify total RNA samples, and reverse transcription was carried out according to the manufacturer’s instructions using the cDNA synthesis kit (cat#679029, Thermo-Scientific^®^, UK). For amplification, 10 µL of Master Mix 2X (Maxima Syber Green/ROX, cat#896415, Thermo-Scientific^®^, UK), 1 µL of each forward and reverse oligo-primers (Macrogen^®^, Rockville, MD, USA), and 7 µL of nuclease-free water (cat#AM9932, Ambion^®^, Naugatuck, CT, USA) were transferred to 96 well plate. A thermal cycler (iQ5 Bio-Rad^®^, Hercules, CA, USA) was set at 95 °C, 40 cycles of denaturation, 60 °C for annealing, and 72 °C for extension. Finally, the 2*^(−ΔΔCt)^ method was employed to calculate the relative mRNA expressions with β-actin (housekeeping gene).

#### 2.4.8. Radiological Examination

Normal and adjuvant-injected hind limbs of rats were subjected to radiological examination. Degenerative changes, such as soft tissue inflammation, osteolysis, and ankylosis of ankle joint, were semi-quantitatively assessed, according to previously devised scoring (0–3) method, using X-ray equipment (KXO-12R, Toshiba^®^, Minato-ku, Japan, set at 200 mAs and 50 KVp) [40,41].

#### 2.4.9. Histological Examination

Hind paw tissues of normal and adjuvant-injected rats were collected, cleaned with normal saline, and stored in a 10% neutral formalin buffered solution. Tissues were dehydrated in graded dilutions of ethanol before being embedded in paraffin. Then, sections of 5–6 µm thickness were sliced with a rotary microtome and mounted on glass slides. Further, these were stained with hematoxylin and eosin (H&E) dyes, and the prepared slides were observed for histopathological changes under a light microscope (IRMECO^®^, Lütjensee, Germany). Images were captured using the TOUPCAM^®^ software package (version x64, 4.11.20805.20220506, ToupTek Photonics Co., Ltd., Hangzhou, China). Semi-quantitative histopathological scores (0–4) indicating bone erosion, tissue inflammation, and inflammatory cell infiltration in paw tissues were evaluated according to the method reported by Zhang et al. [42].

### 2.5. Statistical Analysis

All experimental data were presented as mean ± SD. One-way and two-way ANOVA (wherever appropriate) following post hoc Tukey’s test were applied using GraphPad Prism^®^ software v6.01. A statistical difference of *p* ≤ 0.05 between different groups was considered significant.

## 3. Results

### 3.1. Characterization of Nanoparticles

#### 3.1.1. Zeta Size, Zeta Potential, Polydispersity Index, and Encapsulation Efficiency

**Figure 1:**
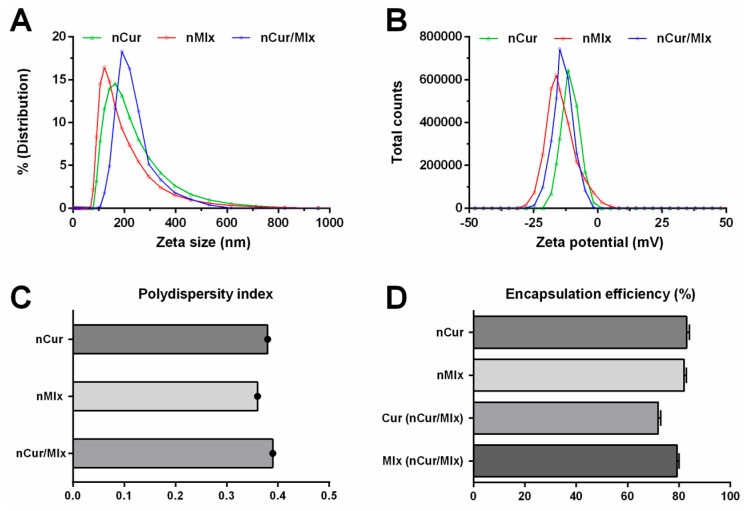
Characterization of curcumin and meloxicam mono and dual-compound-loaded nanoparticles. (**A**) Zeta size, (**B**) zeta potential, (**C**) polydispersity index, and (**D**) encapsulation efficiency of nanoparticles. Cur, curcumin; Mlx, meloxicam; nCur, curcumin-loaded nanoparticles; nMlx, meloxicam-loaded nanoparticles; nCur/Mlx, curcumin plus meloxicam co-loaded nanoparticles.

PLGA nanoparticles encapsulating curcumin (nCur) and meloxicam (nMlx) alone and combined (nCur/Mlx) were subjected to DLS characterization to measure zeta size, zeta potential, and polydispersity index. Figure 1A indicates the 168.13 nm, 122.41 nm, and 193.78 nm zeta sizes of nCur, nMlx, and nCur/Mlx, respectively. A zeta potential of −11.51 mV for nCur, −16.10 for nMlx, and −14.81 for nCur/Mlx was found, as shown in Figure 1B. Meanwhile, the polydispersity index of nCur, nMlx, and nCur/Mlx was 0.38, 0.36, and 0.39, respectively (Figure 1C). The encapsulation efficiencies (EE) were determined through the indirect method, and results that showed the EE of nCur and nMlx was 82.98% and 82.04%, respectively. The nCur/Mlx showed 71.89% EE of curcumin and 79.26% of meloxicam (Figure 1D).

#### 3.1.2. FT-IR and SEM Characterization

**Figure 2:**
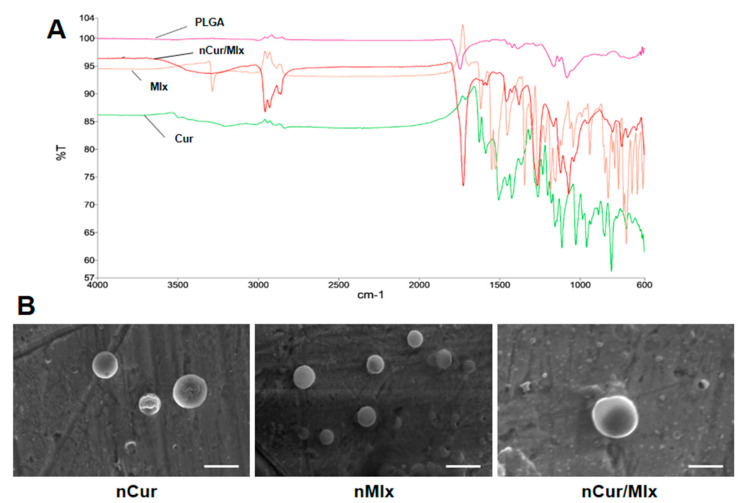
(**A**) FT-IR spectra of PLGA, curcumin, meloxicam, and nCur/Mlx. (**B**) SEM images of nCur, nMlx, and nCur/Mlx. Cur, curcumin; Mlx, meloxicam; nCur, curcumin-loaded nanoparticles; nMlx, meloxicam-loaded nanoparticles; nCur/Mlx, curcumin plus meloxicam co-loaded nanoparticles.

The FT-IR analysis of PLGA, curcumin, meloxicam, and nCur/Mlx was carried out to highlight any polymer–compound interactions (Figure 2A). FT-IR spectrum of nCur/Mlx showed characteristic O-H stretching at 3290 cm^−1^, C-H stretching at 2929 cm^−1^, C=O stretching at 1729 cm^−1^, C=N stretching at 1580 cm^−1^, and C-O stretching in the region of 1400–1100 cm^−1^, which closely resembled the corresponding peaks of PLGA, curcumin, and meloxicam. Figure 2B shows the surface morphology of nCur, nMlx, and nCur/Mlx observed using SEM. All formulations showed nearly smooth-surfaced spherical nanoparticles without significant aggregation or adhesion. A small portion of hollow nanoparticles was also observed.

### 3.2. Effect on Paw Swelling and Arthritic Score

**Figure 3:**
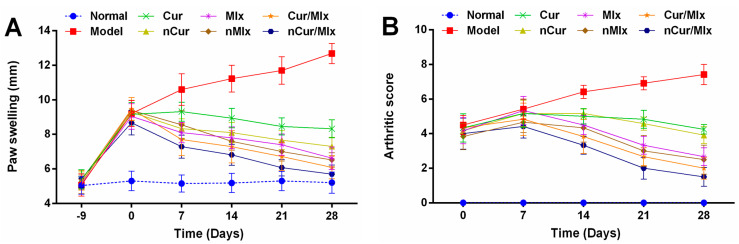
Changes in (**A**) paw swelling and (**B**) arthritic score of curcumin and meloxicam mono and dual-compound-encapsulated nanoparticles at different time intervals of experiment (mean ± SD, *n* = 6). Cur, curcumin; Mlx, meloxicam; Cur/Mlx, curcumin plus meloxicam; nCur, curcumin-loaded nanoparticles; nMlx, meloxicam-loaded nanoparticles; nCur/Mlx, curcumin plus meloxicam co-loaded nanoparticles.

Adjuvant injection into the sub-plantar area of each rat’s paw significantly (*p* ≤ 0.05) induced paw swelling (Figure 3A) and arthritic score (Figure 3B), which were substantially increased from day 0 to 28 of the experiment, as compared to normal group. Maximum paw swelling and arthritic score in the model group were recorded on the 28th day of the experiment. The treatment of arthritic rats with curcumin, meloxicam, curcumin plus meloxicam, nCur, nMlx, and nCur/Mlx demonstrated a gradual reduction in these parameters from day 7 onwards in comparison to the model group (*p* ≤ 0.05). Furthermore, a notable improvement in reducing paw swelling and arthritic score was observed in nCur, nMlx, and nCur/Mlx-treated groups compared to the pure compound-treated groups; however, nCur/Mlx showed the highest attenuating effects among all treatments.

### 3.3. Effect on Body Weight and Immune Organ Index

**Figure 4:**
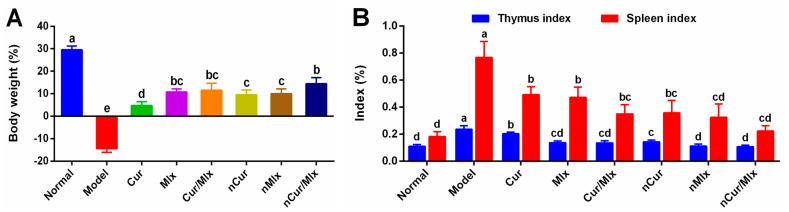
(**A**) Change in body weight and (**B**) immune organ index of thymus and spleen of arthritic rats treated with curcumin and meloxicam mono and dual-compound-loaded nanoparticles after 28 days of experiment. Data were analyzed using one-way ANOVA and Tukey’s test (mean ± SD, *n* = 6). Different alphabets (a–e) indicate significant (*p* ≤ 0.05) differences between groups. Cur, curcumin; Mlx, meloxicam; Cur/Mlx, curcumin plus meloxicam; nCur, curcumin-loaded nanoparticles; nMlx, meloxicam-loaded nanoparticles; nCur/Mlx, curcumin plus meloxicam co-loaded nanoparticles.

Results showed that adjuvant-induced arthritis development resulted in significant (*p* ≤ 0.05) body weight loss (Figure 4A) and an increase in the immune organ index of the thymus and spleen (Figure 4B) compared to the normal group. Meanwhile the administration of treatments including curcumin, meloxicam, curcumin plus meloxicam, as well as PLGA nanoparticles (nCur, nMlx, and nCur/Mlx) for 28 days yielded significant (*p* ≤ 0.05) improvement in body weight and restored the immune organ indices of arthritic rats compared to the model group. Moreover, curcumin co-encapsulation with meloxicam (nCur/Mlx) showed a significantly (*p* ≤ 0.05) enhanced effect on restoring body weight and immune organ indices compared to nCur or nMlx.

### 3.4. Effect on Hematological Parameters

Hematological markers, such as red blood cells (RBCs), hemoglobin (Hb), and white blood cells (WBCs), were determined in normal and treated arthritic rats. The results shown in Table 1 indicate a significant (*p* ≤ 0.05) reduction in RBCs and Hb and an increase in WBCs in the model group in comparison to normal rats. The administration of nCur, nMlx, and nCur/Mlx significantly (*p* ≤ 0.05) ameliorated hematological markers in arthritic rats in comparison to curcumin, meloxicam, and curcumin plus meloxicam-treated groups. In addition, nCur/Mlx revealed significantly (*p* ≤ 0.05) better amelioration of RBCs, Hb, and WBCs as compared to other treated groups.

### 3.5. Effect on Serum Inflammatory Markers

The serum levels of rheumatoid factor (RF), C-reactive protein (CRP), and prostaglandin E2 (PGE2) measured in arthritic rats treated with free compounds and PLGA nanoparticles encapsulating curcumin and meloxicam alone and combined for 28 days are mentioned in Table 2. In comparison to the normal group, arthritis induction resulted in a significant (*p* ≤ 0.05) elevation in RF, CRP, and PGE2 levels in the sera of the model group. Adjuvant-induced arthritic rats treated with curcumin, meloxicam, and curcumin plus meloxicam revealed that curcumin significantly (*p* ≤ 0.05) enhanced the effect of meloxicam and substantially lowered the serum levels of RF, CRP, and PGE2. In addition, the nCur, nMlx, and nCur/Mlx groups showed a significant (*p* ≤ 0.05) difference from pure compound-treated groups, while nCur/Mlx demonstrated the most significant (*p* ≤ 0.05) and highest reduction in the aforementioned serum markers among all treatments.

### 3.6. Effect on Oxidative Stress Parameters

**Figure 5:**
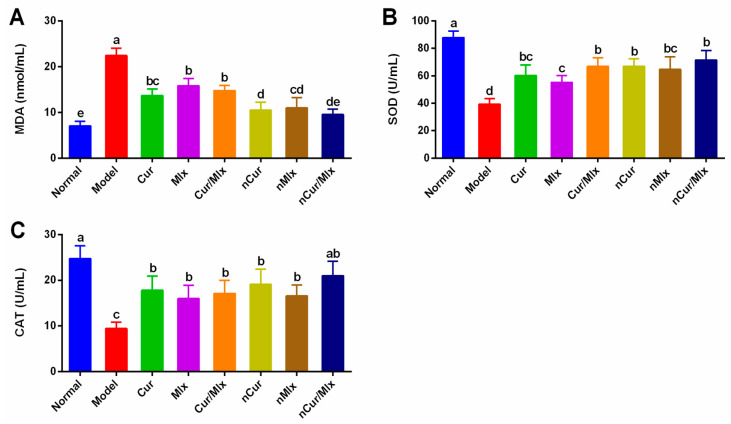
Serum levels of (**A**) MDA, (**B**) SOD, and (**C**) CAT in curcumin and meloxicam mono and co-encapsulated nanoparticle-treated adjuvant-induced arthritic rats after 28 days of the experiment. Data were analyzed using one-way ANOVA and Tukey’s test (mean ± SD, *n* = 6). Different alphabets (a–e) indicate significant (*p* ≤ 0.05) differences between groups. Cur, curcumin; Mlx, meloxicam; Cur/Mlx, curcumin plus meloxicam; nCur, curcumin-loaded nanoparticles; nMlx, meloxicam-loaded nanoparticles; nCur/Mlx, curcumin plus meloxicam co-loaded nanoparticles; MDA, malondialdehyde; SOD, superoxide dismutase; CAT, catalase.

Oxidative stress was assessed in normal, untreated (model), and treated arthritic rats by determining the serum malondialdehyde (MDA) levels and activities of superoxide dismutase (SOD) and catalase (CAT). The results showed that a significant (*p* ≤ 0.05) elevation in MDA and a decrease in SOD and CAT was induced by adjuvant injection, as observed in the model group compared to the normal group (Figure 5). In comparison to free compound-treated groups, a noteworthy (*p* ≤ 0.05) reduction in MDA and an increase in SOD were noticed in arthritic rats treated with nCur, nMlx, and nCur/Mlx. Meanwhile, a non-significant (*p* ≥ 0.05) difference in CAT was seen in curcumin, meloxicam, curcumin plus meloxicam, nCur, and nMlx-treated groups, except for the nCur/Mlx group (*p* ≤ 0.05).

### 3.7. Effect on Inflammatory Cytokines and OPG/RANKL Expressions

**Figure 6:**
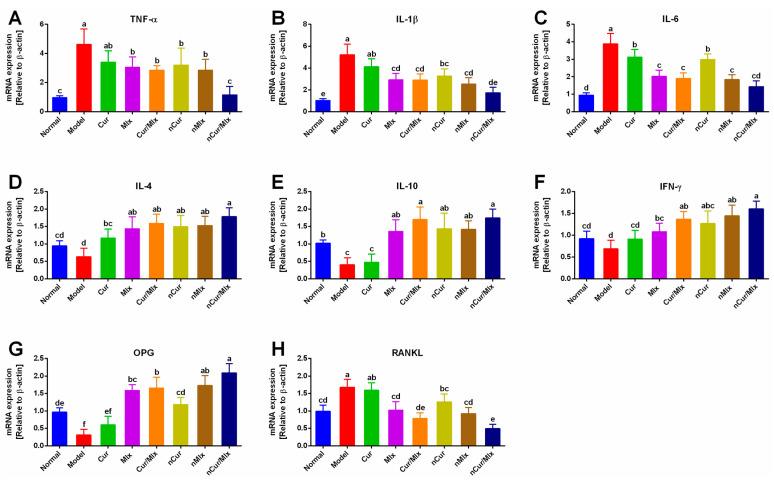
Relative mRNA expressions of pro-/anti-inflammatory cytokines and OPG/RANKL in paw tissue of adjuvant-induced arthritic rats treated with curcumin and meloxicam mono and dual-compound-loaded nanoparticles. (**A**) TNF-α, (**B**) IL-1β, (**C**) IL-6, (**D**) IL-4, (E) IL-10, (**F**) IFN-γ, (**G**) OPG, and (**H**) RANKL. Data were analyzed using one-way ANOVA and Tukey’s test (mean ± SD, *n* = 6). Different alphabets (a–f) indicate significant (*p* ≤ 0.05) differences between groups. Cur, curcumin; Mlx, meloxicam; Cur/Mlx, curcumin plus meloxicam; nCur, curcumin-loaded nanoparticles; nMlx, meloxicam-loaded nanoparticles; nCur/Mlx, curcumin plus meloxicam co-loaded nanoparticles; TNF-α, tumor necrosis factor-α; IL-1β, interleukin-1β; IL-4, interleukin-4; IL-6, interleukin-6; IL-10, interleukin-10; IFN-γ, interferon-γ; OPG, osteoprotegerin; RANKL, receptor activator of nuclear factor kappa-Β ligand.

The qRT-PCR analysis was conducted to assess the mRNA expression levels of inflammatory mediators, including tumor necrosis factor-α (TNF-α), interleukins (IL-1β, IL-6, IL-4, and IL-10), interferon-γ (IFN-γ), as well as osteoprotegerin (OPG) and receptor activator of nuclear factor kappa-Β ligand (RANKL) in the paw tissue of arthritic rats treated with curcumin and meloxicam mono and dual-compound-loaded nanoparticles (Figure 6). The results showed that the adjuvant induced a significant (*p* ≤ 0.05) up-regulation of pro-inflammatory cytokines, such as TNF-α, IL-1β, and IL-6 expressions (Figure 6A–C), and down-regulated the expression levels of anti-inflammatory cytokines, including IL-4, IL-10, and IFN-γ, in the model group, as shown in Figure 6D–F. In addition, the significantly (*p* ≤ 0.05) decreased expression of OPG and increased RANKL expression were found in the model group (Figure 6G,H). As compared to the model group, the administration of curcumin and meloxicam mono (nCur and nMlx) and dual-compound-loaded nanoparticles (nCur/Mlx) significantly (*p* ≤ 0.05) suppressed the expressions of pro-inflammatory cytokines (TNF-α, IL-1β, and IL-6), promoted the anti-inflammatory cytokines (IL-4, IL-10, and IFN-γ), and modulated the expressions of OPG and RANKL in contrast to the model group. Moreover, it was also observed that nCur/Mlx exhibited higher anti-arthritic activity among all treatments, evidenced by the significant (*p* ≤ 0.05) modulation of expression levels of TNF-α, IL-1β, IL-6, IL-4, IL-10, IFN-γ, OPG, and RANKL in the paw tissues of arthritic rats.

### 3.8. Gross and Radiological Findings

**Figure 7:**
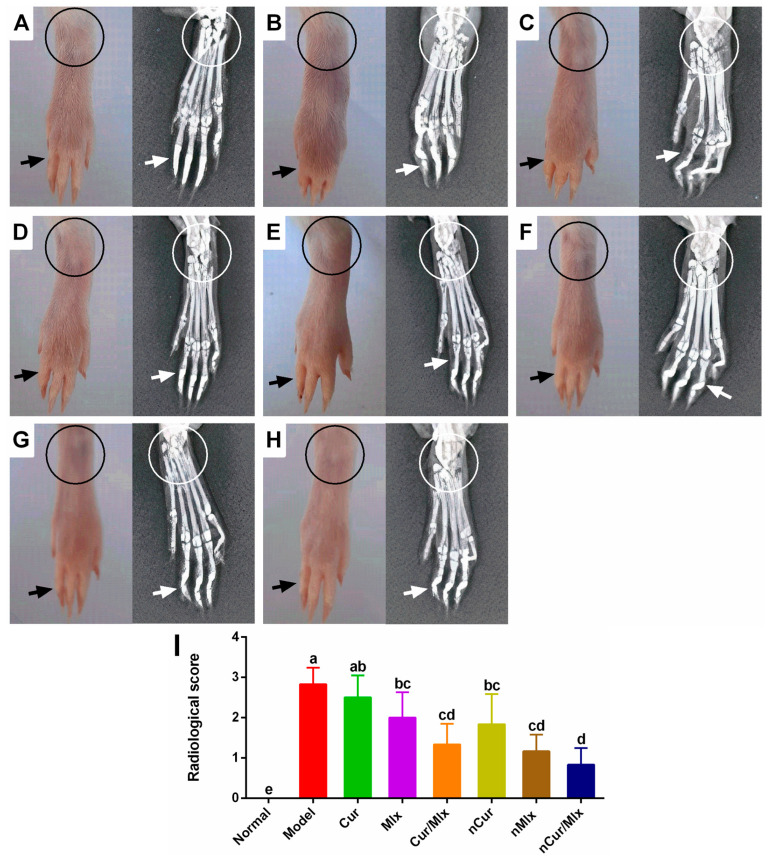
Macroscopic and radiological images of adjuvant-induced arthritic rats treated with curcumin and meloxicam mono and co-encapsulated nanoparticles for 28 days. (**A**) Normal rats, (**B**) model group, and arthritic rats treated with (**C**) Cur, curcumin, (**D**) Mlx, meloxicam, (**E**) Cur/Mlx, curcumin plus meloxicam, (**F**) nCur, curcumin-loaded nanoparticles, (**G**) nMlx, meloxicam-loaded nanoparticles, and (**H**) nCur/Mlx, curcumin plus meloxicam co-loaded nanoparticles. Circle: joint ankylosis and osteolysis; arrow: tissue inflammation. (**I**) Radiological scores were analyzed using one-way ANOVA and Tukey’s test and presented as mean ± SD (*n* = 6). Different alphabets (a–e) indicate significant (*p* ≤ 0.05) differences between groups.

The hind limbs of normal and adjuvant-injected rats treated with curcumin, meloxicam, and curcumin plus meloxicam and their PLGA-loaded nanoparticles were subjected to macroscopic and radiological examinations (Figure 7). Compared to normal rats, the model group indicated tissue inflammation and joint ankylosis and osteolysis that were consistent with paw swelling and arthritic score. The treatment of arthritic rats with curcumin and meloxicam demonstrated an inhibition of tissue inflammation and joint degeneration; meanwhile, curcumin potentiated the anti-arthritic effect of meloxicam. nCur and nMlx yielded mild joint ankylosis with less tissue swelling. Furthermore, nearly normal radiological features were observed in nCur/Mlx-treated rats.

### 3.9. Histopathological Findings

**Figure 8:**
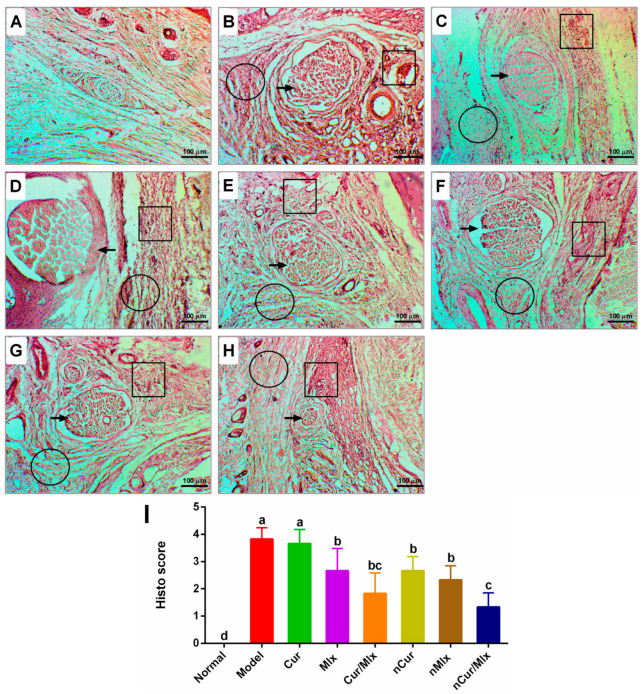
Paw histology images of mono and dual-compound-loaded nanoparticles administered adjuvant-induced arthritic rats (magnification ×100). (**A**) Normal rats, (**B**) model group, and arthritic rats treated with (**C**) Cur, curcumin, (**D**) Mlx, meloxicam, (**E**) Cur/Mlx, curcumin plus meloxicam, (**F**) nCur, curcumin-loaded nanoparticles, (**G**) nMlx, meloxicam-loaded nanoparticles, and (**H**) nCur/Mlx, curcumin plus meloxicam co-loaded nanoparticles. Box: inflammatory cell infiltration; circle: tissue edema; arrow: bone erosion. (**I**) Histopathological scores were analyzed using one-way ANOVA and Tukey’s test and presented as mean ± SD (*n* = 6). Different alphabets (a–d) indicate significant (*p* ≤ 0.05) differences between groups.

The histological examination of paw tissues of normal, model (untreated), and treated arthritic rats was performed at the end of the experiment to observe the degenerative changes induced by the adjuvant (Figure 8). Normal paw histo-structures were observed in the normal group. In the model group, adjuvant injection caused extensive bone erosion, infiltration of inflammatory cells, and tissue edema and necrosis. Arthritic rats treated with curcumin, meloxicam, and curcumin plus meloxicam exhibited moderately reduced degenerative changes. Also, curcumin in combination with meloxicam showed a better effect than curcumin or meloxicam alone. PLGA encapsulation markedly enhanced the pharmacological activities of curcumin, meloxicam, and curcumin plus meloxicam, as relatively mild histopathological changes were observed in nCur, nMlx, and nCur/Mlx-treated groups.

## 4. Discussion

RA is one of the prevalent autoimmune diseases that affect a significant proportion of the world’s population. It is manifested by distinguishing symptoms, such as swelling, pain, and stiffness of joints, along with other clinical complications [1,4,5]. Nanoformulations have gained popularity among researchers due to their improved pharmacological and therapeutic effectiveness, which expand their utility in biomedical research [24,43,44].

In this study, PLGA used in the synthesis of curcumin and meloxicam alone (nCur and nMlx) and combined (nCur/Mlx) nanoparticles is acid-terminated and functionalized with carboxylic acid groups, as it yields small-sized and stable nanoparticles. The deprotonation of free carboxylic acid groups in PLGA imparts negative zeta potential to nanoparticles. Also, small-sized nanoparticles have higher zeta potential values than larger ones, resulting in the enhanced stability of nanoparticles [45]. The results showed that the zeta size of nCur, nMlx, and nCur/Mlx ranged from 122.41 nm to 193.78 nm (Figure 1A), the zeta potential ranged from −16.10 mV to −11.51 mV (Figure 1B), and the polydispersity index was found to be less than 0.40 (Figure 1C), which indicated the uniformity and stability of all prepared nanoparticles. The zeta size of synthesized nanoparticles was within the optimal size range for nano-drug delivery formulations. Nanoparticles less than 20 nm can be filtered out by the renal system, while those above 200 nm can be removed by the reticuloendothelial system [46].

Encapsulation efficiency (EE%) is an essential parameter that must be taken into onsideration when evaluating nanoparticles since it primarily influences drug release patterns as well as their in vivo implementation [47]. PLGA prevents entrapped drug molecules from diffusing to the external surface, which results in a considerable rise in EE [48]. The EE of nanoparticles can be measured directly or indirectly [49]. The indirect method was employed in this current study, and the amount of free compounds (curcumin and meloxicam) in the supernatant following the centrifugation of nanoparticles was determined and used to assess EE. Results demonstrated that nCur, nMlx, and nCur/Mlx had a high EE (>70%), as measured by an indirect technique (Figure 1D).

FT-IR is a key analytical method that should be thoroughly studied during nanoparticle synthesis to find out any physical and chemical interactions that might occur between the polymeric material and encapsulated compound, as well as to assure compatibility between all components of nanoparticles [50]. The FT-IR spectrum of nCur/Mlx demonstrated the presence of functional groups of PLGA and free compounds, including curcumin and meloxicam, indicating that no chemical interaction occurred between polymer and loaded compounds (Figure 2A). Further, SEM results showed spherical and smooth-surfaced nanoparticles with limited particle size dispersion. The surface morphology of nCur, nMlx, and nCur/Mlx showed no significant differences (Figure 2B). SEM size distributions were substantially identical to DLS results. A few nanoparticles with porous surfaces were also discovered, which is anticipated to aid medication solubility and subsequent polymer breakdown [51].

Complete Freund’s adjuvant-induced arthritis animal model is commonly employed because it induces extensive tissue inflammation and joint remodeling, along with body weight loss and biochemical indications, which are similar to the symptoms of human RA. Also, this model is utilized for studying the molecular mechanisms involved in inflammation and auto-immune diseases, such as rheumatoid arthritis [27,52]. Like human RA, the progression of adjuvant-induced arthritis can be divided into phases, such as the induction phase with no visible confirmation of synovitis, followed by early stage synovitis, and final stage synovitis with joint damage. These stages should be alleviated by effective anti-rheumatic medications. The production of PGs and inflammation development are the main events in primary arthritis, whereas autoantibodies are formed in the subsequent phases. The release of numerous inflammatory mediators is critical in the development of pain, joint inflammation, bone deformation, and joint dysfunction [4,6,53,54].

In this study, we utilized adjuvant-induced arthritis in rats to investigate the anti-arthritic activity of nCur/Mlx in comparison to nCur, nMlx and pure compounds. The results showed that adjuvant injection in the rats’ paws induced a gradual increase in paw inflammation and arthritic score, which were at a maximum on the 28th day of the experiment (Figure 3A,B). Paw edema and arthritic score are linked to immune cell infiltration in the inflammatory site and an increase in vascular permeability [55]. These parameters are simple to assess the efficiency of anti-arthritic agents. A reduction in paw swelling and arthritic score exhibits a decrease in the outflow of inflammatory mediators and indicates the anti-inflammatory action of treatment. In this present study, paw swelling persisted in the model group for 28 days due to continuous cellular invasion and edema. However, in pure compounds and nanoparticles, including nCur, nMlx, and nCur/Mlx-treated groups, the maximum paw swelling and arthritic score were recorded on day 0 (−9), after which the inflammation began to subside. It was also found that nCur/Mlx demonstrated significant (*p* ≤ 0.05) improvement in reducing these parameters compared to nCur and nMlx.

RA is associated with a decrease in body weight, which is known as rheumatoid cachexia. It might be related to the severity of joint inflammation, assuming that the weight loss is caused by disease-related stress, hyper-algesia, malabsorption, and muscle proteolysis [56]. The administration of anti-inflammatory agents may help to reduce pain and inflammation and restore normal gastrointestinal function. Our results showed a significant (*p* ≤ 0.05) body weight loss in the model group, which was restored in the treated groups (Figure 4A). Furthermore, immune hyper-functioning can be evidenced by an enlarged thymus and spleen in the model group, as reported in previous studies [57,58]. Arthritic rats treated with pure compounds and nanoparticles revealed immune suppression by significantly (*p* ≤ 0.05) restoring thymus and spleen indices (Figure 4B).

The adjuvant-induced arthritis model of rats is also employed to investigate hematological and biochemical abnormalities. The reduction in RBCs and Hb in arthritic rats resulted in anemia, which is caused by erythrocyte disruption, bone marrow failure, and decreased erythropoietin [59]. Furthermore, a significant (*p* ≤ 0.05) increase in WBCs was seen in the model group, indicating the immune system activation [60]. PLGA nanoparticles encapsulating curcumin and meloxicam alone (nCur and nMlx) and combined (nCur/Mlx) significantly (*p* ≤ 0.05) raised RBCs and Hb and reduced WBCs in arthritic rats compared to the model group (Table 1), resulting in a delay in anemia onset and immunosuppression. Systemic markers such as RF and CRP are useful in determining incidence, progression, and severity of RA [5,61]. In this present study, significantly (*p* ≤ 0.05) elevated serum levels of RF and CRP in the model group indicated active systemic inflammation and RA progression. A significant (*p* ≤ 0.05) decrease in RF and CRP levels in arthritic rats treated with nCur, nMlx, and nCur/Mlx showed a reduction in systemic inflammation (Table 2), which might be linked to TNF-α and IL-6 inhibition [62]. In RA, COX-2 activation generates PGs, such as PGE2, which cause joint pain and inflammation. Pro-inflammatory cytokines, such as TNF-α and IL-1β, can stimulate the generation of PGE2 from synovial fibroblasts and chondrocytes, as observed in arthritic rats. PGE2 further interacts with immune cells and promotes the release of inflammatory mediators [63]. Our results showed that the administration of nCur, nMlx, and nCur/Mlx resulted in a significant (*p* ≤ 0.05) inhibition of PGE2 in arthritic rats (Table 2), which is similar to a prior study [64]. In the current investigation, nCur/Mlx exhibited promising anti-arthritic activity by improving arthritis symptoms, hematological markers, and biochemical changes in contrast to nCur and nMlx, which was comparable to prior studies [52,64,65].

Cellular oxidative damage is caused by elevated levels of lipid peroxides and excessive ROS generation, both of which are believed to be critical mediators of oxidative injury. Other than the protective activity of antioxidant enzymes, increased ROS formation causes a loss of homeostatic balance, resulting in the impairment of antioxidant defense mechanisms [66,67]. These circumstances also decrease the activity of SOD, which detoxifies peroxide and superoxide radicals, as well as disturbs the hydrogen peroxide detoxification potential of CAT [68,69]. Previous studies showed that increased ROS buildup accelerates the pathogenic processes implicated in RA. Increased ROS production in RA leads to a decrease in antioxidant functions, which further aggravates RA development by increasing oxidative stress and necrosis of cells [8]. In arthritic rats supplemented with mono and dual-compound-loaded nanoparticles, the status of lipid peroxidation (MDA) was significantly (*p* ≤ 0.05) decreased, while the status of antioxidant enzymes, such as SOD and CAT, was significantly (*p* ≤ 0.05) elevated (Figure 5A–C). The results confirmed the improvement in the antioxidant activity of nanoparticles, particularly of nCur/Mlx compared to nCur or nMlx.

Pro-inflammatory cytokines, including TNF-α, IL-1β, and IL-6, play an important role in the pathogenesis of RA, and these pro-inflammatory mediators can be abundantly found in adjuvant-injected rats. These cytokines induce local and subsequent systemic inflammation and cause the stimulation of osteoclasts, all of which lead to cartilage damage, bone resorption, and the degradation of extracellular matrix. Synoviocytes serve as a reservoir for these inflammatory mediators [7,11,63,64]. TNF-α amplifies inflammation by stimulating synovial fibroblasts, which increases the cellular adhesion of mediators and leukocyte movement, subsequently resulting in joint injury. IL-1β governs cartilage degradation and bone resorption as well as the generation of PGE2 and nitric oxide (NO), whereas PGE2 induces fever and stimulates pain receptors. IL-6 promotes angiogenesis and also involves inflammation induction [69]. The results of qRT-PCR analysis demonstrated that nCur/Mlx significantly (*p* ≤ 0.05) inhibited the expression of pro-inflammatory cytokines such as TNF-α, IL-1β, and IL-6 when compared to nCur and nMlx (Figure 6A–C). Thus, nCur/Mlx showed beneficial effects in reducing pro-inflammatory cytokine-mediated inflammatory and structural changes.

IL-4 and IL-10 are key immunomodulatory cytokines that stimulate Th2 cell production while inhibiting Th1 response, hence limiting autoimmune disorders. IL-4 inhibits Th1 production while promotes Th2 cell production. Meanwhile, IL-10 suppresses Th1 cell-mediated generation of cytokines (IL-1β, IFN-γ, and TNF-α) and inhibits IL-18 production. It also decreases the activity of antigen-presenting cells and maintains joint integrity [70,71]. Furthermore, IFN-γ improves the inflammatory response by decreasing Th17 cell development and osteoclasts [72]. Thus, IL-4, 1L-10, and IFN-γ inhibit RA-linked inflammation, bone degeneration, and cartilage breakdown. The current study found that curcumin and meloxicam encapsulated mono (nCur and nMlx) and co-loaded nanoparticles (nCur/Mlx) treated arthritic rats exhibited significant (*p* ≤ 0.05) overexpression of IL-4, 1L-10, and IFN-γ than pure compound-treated groups (Figure 6D–F).

OPG/RANKL is assumed to be critical in bone metabolism-regulating mechanism that affects osteoclastogenesis. OPG inhibits bone resorption by blocking RANKL from attaching to its receptors, while RANKL promotes bone resorption via interacting with its receptors expressed on osteoclasts [73,74]. The current investigation found that curcumin co-encapsulation with meloxicam (nCur/Mlx) significantly (*p* ≤ 0.05) up-regulated the mRNA expression of OPG and down-regulated RANKL expression in adjuvant-induced arthritic rats, as compared to monotherapy (Figure 6G,H). Thus, the findings of qRT-PCR analysis suggest that curcumin potentiated the anti-arthritic activity of meloxicam co-encapsulated nanoparticles (nCur/Mlx) by inhibiting the pro-inflammatory cytokines while up-regulating anti-inflammatory cytokines, and ultimately modulating the OPG/RANKL expressions.

Radiological examination is crucial for validating disease recurrence and determining the status of disease. In RA, bone loss is caused by decreased bone conformation and increased bone resorption [75]. Therefore, the radiological examination of adjuvant-induced arthritis in rats revealed the inflammation of soft tissue, joint deformity with resorption, bone erosion, narrower joint gaps (ankylosis), and cartilage degradation in the model group, as observed in an earlier study [51]. According to the radiological findings of treated arthritic rats, pure compound and PLGA nanoparticle-encapsulating curcumin and meloxicam alone and in combination, i.e., nCur, nMlx, and nCur/Mlx, significantly inhibited arthritis-linked tissue inflammation and bone damage of the ankle joint, which may be associated with a decrease in the production of inflammatory mediators. These damaging alterations were slightly decreased after therapy with curcumin and meloxicam. There was a significant decrease in the joint inflammation, cartilage degradation, and bone erosion in curcumin plus meloxicam, nCur, and nMlx therapy groups. However, the results also revealed that nCur/Mlx had a significant ameliorative effect on joint and cartilage, which was comparable to the normal group (Figure 7A–I). Thus, among all treatments, curcumin potentially improved the anti-arthritic activity of meloxicam co-loaded nanoparticles (nCur/Mlx), which effectively reduced the inflamed joints, most likely due to improved penetration and significant accumulation at the afflicted tissue. Our results are consistent with a previous study [76].

RA primarily affects the synovium, inducing joint swelling, which could be observed through histopathological studies. Inflammatory mediators such as NF-kB and cytokines (TNF-α, IL-1β, IL-6, and PGE2) are involved in the development of RA symptoms. The release of these inflammatory mediators by inflammatory cells during the attack on the synovial membrane causes bone and joint damage, hyperplasia of synovial membrane, and pannus formation [77]. The histological findings of our study corroborated the biochemical, gene expression, and radiological results. The histological score demonstrated that inflammation was markedly (*p* ≤ 0.05) suppressed by the administration of curcumin, meloxicam, curcumin plus meloxicam, nCur, nMlx, and nCur/Mlx (Figure 8A–I). Results also revealed that mono (nCur, nMlx) and dual-compound (nCur/Mlx)-loaded nanoparticles significantly (*p* ≤ 0.05) decreased the infiltration of inflammatory cells and vascularity, resulting in less tissue edema when compared to pure compound-treated groups. Furthermore, as compared to mono compound-loaded nanoparticles, nCur/Mlx resulted in a significant (*p* ≤ 0.05) alleviation of these histological abnormalities. Our findings were supported by previous studies [78].

## 5. Conclusions

In this present study, we successfully synthesized curcumin and meloxicam co-loaded PLGA nanoparticles of suitable zeta size, encapsulation efficiency, and morphology using the solvent evaporation method. In vivo, our study demonstrated that curcumin significantly potentiated the anti-arthritic activity of meloxicam co-encapsulated PLGA nanoparticles (nCur/Mlx) in adjuvant-induced arthritic rats, evident from the significant restoration of physical parameters and immune organ indices and the attenuation of serum inflammatory markers (RF, CRP, and PGE2) and oxidative stress biomarkers (MDA, SOD, and CAT). Moreover, nCur/Mlx showed a significant modulation of pro-inflammatory cytokines (TNF-α, IL-1β, and IL-6), anti-inflammatory cytokines (IL-4, IL-10, and IFN-γ), OPG, and RANKL expressions in paw tissue compared to monotherapy. Radiological and histopathological findings also supported the biochemical and gene expression results. Thus, It can be concluded that nCur/Mlx could be a novel therapeutic approach to manage rheumatoid arthritis.

## Figures and Tables

**Table 1:** Hematological parameters of curcumin and meloxicam mono and co-encapsulated nanoparticle-treated adjuvant-induced arthritic rats.

Parameters	Groups							
	Normal	Model	Cur	Mlx	Cur/Mlx	nCur	nMlx	nCur/Mlx
RBCs (10^6^/µL)	11.12 ± 1.36 ^a^	4.71 ± 0.57 ^d^	7.89 ± 0.61 ^bc^	7.16 ± 0.44 ^c^	7.27 ± 0.51 ^c^	8.96 ± 1.11 ^b^	8.28 ± 0.62 ^bc^	9.22 ± 1.05 ^b^
Hb (g/dL)	13.64 ± 0.99 ^a^	6.06 ± 0.66 ^d^	9.13 ± 1.34 ^c^	8.56 ± 0.73 ^c^	9.18 ± 1.19 ^c^	11.04 ± 0.91 ^b^	9.89 ± 0.97 ^bc^	11.61 ± 0.91 ^b^
WBCs (10^3^/µL)	8.11 ± 0.48 ^e^	17.36 ± 1.27 ^a^	13.72 ± 1.13 ^b^	12.16 ± 1.15 ^bc^	10.61 ± 1.84 ^cd^	11.32 ± 1.61 ^bc^	10.57 ± 1.01 ^cd^	9.04 ± 0.82 ^de^

Data were analyzed using one-way ANOVA and Tukey’s test (mean ± SD, *n* = 6). Different alphabets (a–e) indicate significant (*p* ≤ 0.05) differences between groups. Cur, curcumin; Mlx, meloxicam; Cur/Mlx, curcumin plus meloxicam; nCur, curcumin-loaded nanoparticles; nMlx, meloxicam-loaded nanoparticles; nCur/Mlx, curcumin plus meloxicam co-loaded nanoparticles; RBCs, Red blood cells; Hb, hemoglobin; WBCs, white blood cells.

**Table 2:** Serum levels of inflammatory markers in adjuvant-induced arthritic rats treated with curcumin and meloxicam mono and co-encapsulated nanoparticles.

Parameters	Groups							
	Normal	Model	Cur	Mlx	Cur/Mlx	nCur	nMlx	nCur/Mlx
RF (IU/L)	4.64 ± 0.53 ^f^	49.78 ± 3.13 ^a^	41.93 ± 2.01 ^b^	27.84 ± 3.15 ^c^	22.99 ± 2.93 ^d^	29.04 ± 1.96 ^c^	18.52 ± 1.57 ^e^	14.69 ± 2.74 ^e^
CRP (mg/L)	1.73 ± 0.19 ^f^	8.39 ± 0.59 ^a^	6.11 ± 0.44 ^b^	4.56 ± 0.36 ^c^	4.48 ± 0.21 ^cd^	5.12 ± 0.45 ^c^	3.80 ± 0.31 ^d^	2.87 ± 0.34 ^e^
PGE2 (pg/mL)	131.05 ± 3.74 ^f^	773.64 ± 31.18 ^a^	501.07 ± 38.87 ^b^	362.60 ± 24.62 ^d^	338.43 ± 24.02 ^d^	422.14 ± 40.41 ^c^	264.82 ± 39.98 ^e^	233.92 ± 18.66 ^e^

Data were analyzed using one-way ANOVA and Tukey’s test (mean ± SD, *n* = 6). Different alphabets (a–f) indicate significant (*p* ≤ 0.05) differences between groups. Cur, curcumin; Mlx, meloxicam; Cur/Mlx, curcumin plus meloxicam; nCur, curcumin-loaded nanoparticles; nMlx, meloxicam-loaded nanoparticles; nCur/Mlx, curcumin plus meloxicam co-loaded nanoparticles; RF, rheumatoid factor; CRP, C-reactive protein; PGE2, prostaglandin E2.

## Data Availability

The data presented in this study are available within the article.
